# Multiscale Rotated Bounding Box-Based Deep Learning Method for Detecting Ship Targets in Remote Sensing Images

**DOI:** 10.3390/s18082702

**Published:** 2018-08-17

**Authors:** Shuxin Li, Zhilong Zhang, Biao Li, Chuwei Li

**Affiliations:** ATR National Key Laboratory, National University of Defense Technology, Changsha 410073, China; lishuxin@nudt.edu.cn (S.L.); libiao_cn@163.com (B.L.); lichuwei17@nudt.edu.cn (C.L.)

**Keywords:** multiscale rotated bounding box, deep learning, ship detection, remote sensing image

## Abstract

Since remote sensing images are captured from the top of the target, such as from a satellite or plane platform, ship targets can be presented at any orientation. When detecting ship targets using horizontal bounding boxes, there will be background clutter in the box. This clutter makes it harder to detect the ship and find its precise location, especially when the targets are in close proximity or staying close to the shore. To solve these problems, this paper proposes a deep learning algorithm using a multiscale rotated bounding box to detect the ship target in a complex background and obtain the location and orientation information of the ship. When labeling the oriented targets, we use the five-parameter method to ensure that the box shape is maintained rectangular. The algorithm uses a pretrained deep network to extract features and produces two divided flow paths to output the result. One flow path predicts the target class, while the other predicts the location and angle information. In the training stage, we match the prior multiscale rotated bounding boxes to the ground-truth bounding boxes to obtain the positive sample information and use it to train the deep learning model. When matching the rotated bounding boxes, we narrow down the selection scope to reduce the amount of calculation. In the testing stage, we use the trained model to predict and obtain the final result after comparing with the score threshold and nonmaximum suppression post-processing. Experiments conducted on a remote sensing dataset show that the algorithm is robust in detecting ship targets under complex conditions, such as wave clutter background, target in close proximity, ship close to the shore, and multiscale varieties. Compared to other algorithms, our algorithm not only exhibits better performance in ship detection but also obtains the precise location and orientation information of the ship.

## 1. Introduction

Ships are important maritime targets and monitoring the ship target is valuable in civil and military applications [1]. With the development of optical remote sensing, the resolution of remote sensing images has gradually increased, enabling the detection and recognition of ship targets. A conventional approach to interpret the optical remote sensing images relies on human expertise, which, however, faces difficulty in satisfying the need of processing mass image data. Therefore, it is necessary to study the problems involved in ship detection.

There are three difficulties in detecting ships from the optical remote sensing images. First, ships vary in appearance and size. The base shape of the ship target is long spindle, but ships of different categories have different scales, aspect ratios, and appearances. Second, different ship targets have different orientations. As remote sensing images are captured above the target from the sky platform, ship targets have many orientations, which make ship detection harder. Third, there is complex background clutter around a ship target, such as wave interference, ships in close proximity, and land background when the ships are close to the shore. There are two conventional approaches for detecting ships. The first is to detect the ship target through heuristic artificially designed multistep methods [1,2,3,4]. Relying on the difference between the ship and the background, these algorithms analyze the region geometric features, such as aspect ratio and scale, to decide in which region the ship target is after segmentation of ocean and land. However, artificially designed steps result in a lack of robustness. The other methods [5,6,7,8] rely on machine learning to train the classifier using features extracted from positive and negative samples. When testing a new image, sliding windows or regions of interest which is produced by a pre-detection method, such as Selective Search, are sent to the classifier to predict the class score, and then, a threshold is set to obtain the final detection result. Using a large scale of positive and negative samples to train the classifier, the performance of the detector is made more robust in complex backgrounds, which is usually used in target verification stage [9]. However, most methods use horizontal bounding boxes to detect the target, which is not suitable for more complex conditions such as targets in close proximity and when the ship is close to the shore.

Recently, deep learning-based methods have made significant progress in computer vision applications. Combining a large-scale dataset and high-performance computing hardware GPUs, deep learning methods have undergone a leap in development in areas such as target detection [10,11,12,13,14], target classification [15,16], and semantic segmentation [17,18]. Deep learning-based detection methods exhibit high performance in detecting common objects in daily images. For example, the Faster R-CNN algorithm [12] adopts a two-stage pipeline to detect objects. The first stage produces the regions of interest from the feature maps extracted through a pretrained network, and the second stage selects the features of regions of interest from the shared feature maps and predicts a more precise classification and localization. The SSD algorithm [13] uses end-to-end training networks to predict the class name of the target and regress the bounding box information from multiscale feature maps, which is produced by the hierarchical down sampling structure of deep network. Combined with the strategy of data augmentation, the performance of SSD is close to that of the Faster R-CNN algorithm and SSD exhibits faster detection than Faster R-CNN. Though exhibiting good performance, the above two algorithms use a horizontal bounding box to detect objects, which is not suitable for rotated objects in close proximity.

**Figure 1:**
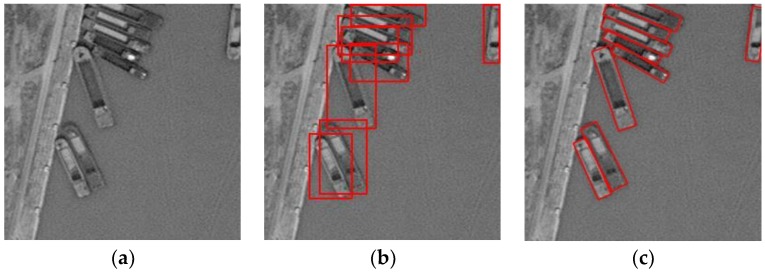
Comparison of target with horizontal and rotated bounding boxes. (**a**) Input image, (**b**) targets with horizontal bounding boxes, and (**c**) targets with rotated bounding boxes.

In the area of ship detection in remote sensing images, there are also deep learning-based methods [19,20,21,22,23], which use deep neural networks to extract robust features from the image. However, most of them are also based on a horizontal bounding box. Recently, deep learning-based methods using a rotated bounding box have been proposed to detect rotated remote sensing targets. Liu et al. proposed a DRBox algorithm [24] to combine the rotated bounding box with deep learning methods to realize rotation-invariant detection. This algorithm can effectively detect airplane, vehicle, and ship targets in remote sensing images and output the precise location and orientation information of the target. However, the approximate calculation in matching the rotated bounding boxes makes the performance unstable. Xia et al. proposed an improved Orientated Bounding Box based (OBB) Faster R-CNN algorithm [25] using eight parameters, which include the coordinates of the four corner points $\left(x_{1},y_{1},x_{2},y_{2},x_{3},y_{3},x_{4},y_{4}\right)$, to record the rotated bounding box based on the Faster R-CNN algorithm. The algorithm can effectively detect the oriented targets, but the predicted bounding box sometimes becomes deformed as there is no constraint to maintain the rectangular shape of the box. Figure 1 shows a comparison of the target with horizontal and rotated bounding boxes. When using horizontal bounding boxes to label targets in close proximity, it is difficult to distinguish the rotated targets, while using rotated boxes, they can be clearly distinguished.

This paper proposes a rotated bounding box-based deep learning algorithm to detect ship targets in remote sensing images. We aim to detect rotated ships in a complex background more effectively. Based on the deep networks, we combine the rotated bounding box with the convolutional feature maps to simultaneously predict the location and orientation of the ship target. When labeling the ship targets, we add an angle parameter and use five parameters, including two center point coordinates, height, width, and angle of the box, to record a rotated box, which will limit the box to have a rectangular shape. In the training stage, we first set prior rotated boxes at each location of feature maps produced by networks and match them with the ground-truth boxes to obtain the positive sample information. Through the range control strategy, we narrow down the selection scope and the amount of calculation required for the matching is reduced. In the testing stage, the test image is sent to the network to predict the class, location, and orientation information of the target. Through comparing the scores with the threshold and post-processing of nonmaximum suppression, we can obtain the final detection and location result. This paper has the following contributions. (1) Combining the rotated bounding boxes with the deep learning methods to simultaneously detect and locate the rotated ship targets in a complex background, (2) adding an angle parameter to the original four parameters to record a rotated bounding box to maintain the rectangular shape of the box, and (3) using a range control strategy to calculate the matching between the prior rotated boxes and ground-truth boxes to reduce the amount of calculation.

## 2. Method to Label the Rotated Targets

To label the rotated targets, we need to add a parameter, besides the original four parameters, to record the angle information of the rotated target. The original labeling method records the coordinates of the top-left and bottom-right corners to uniquely determine a horizontal bounding box. In the deep learning object detection methods, the parameters are transformed as $\left(x_{c},y_{c},w,h\right)$, where $\left(x_{c},y_{c}\right)$ is the coordinate of the center point, w is the width of the box, and h is the height of the box. The four parameters can better explain the feature of the box and ensure that the detection algorithm converges effectively.

**Figure 2:**
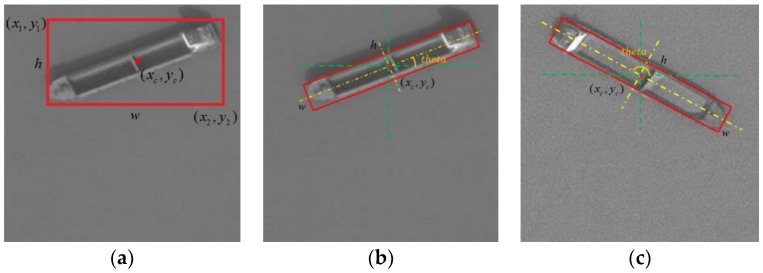
Comparison of parameters used in horizontal and rotated bounding boxes. (**a**) Parameters used in horizontal bounding boxes and (**b**,**c**) parameters used in rotated bounding boxes.

Based on these four parameters, we add another angle parameter theta to uniquely determine a rotated bounding box $\left(x_{c},y_{c},w,h,theta\right)$, where $\left(x_{c},y_{c}\right)$ is still the coordinate of the center point; h represents the long side of the box; w represents the short side of the box, which is perpendicular to h; and theta represents the angle between *h* in the upward direction and the horizontal right direction, and is in the range of [0,180) degrees. The labeling parameters are shown in Figure 2.

## 3. Multiscale Rotated Bounding Box-Based Deep Learning Detection Model

Our algorithm is based on deep learning methods and uses the multiscale rotated bounding boxes to detect and locate the ships precisely. This section gives a comprehensive explanation of the algorithm from the perspectives of network structure, design of the key parts, and implementation details.

### 3.1. Network Structure

**Figure 3:**
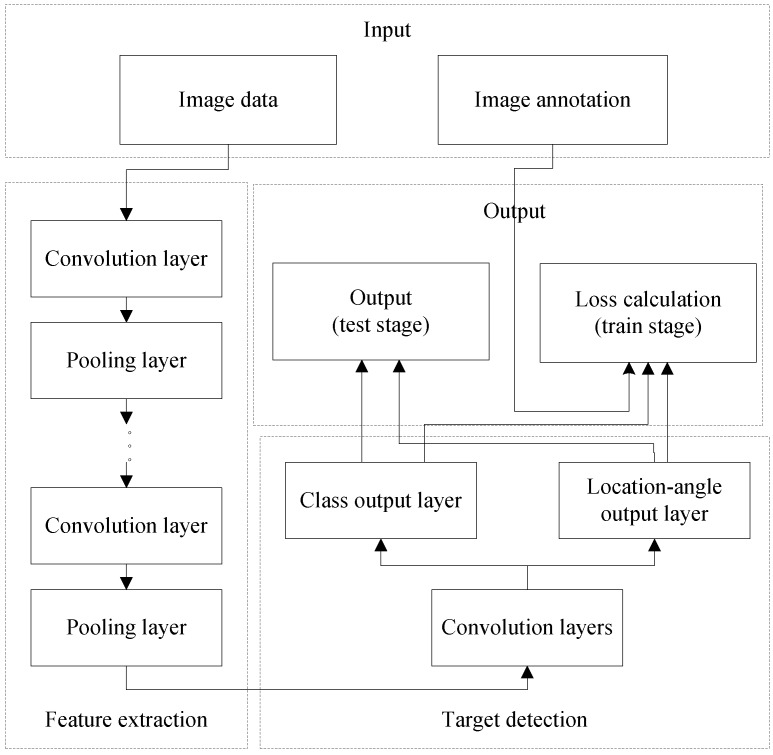
Diagram of network structure.

**Figure 4:**
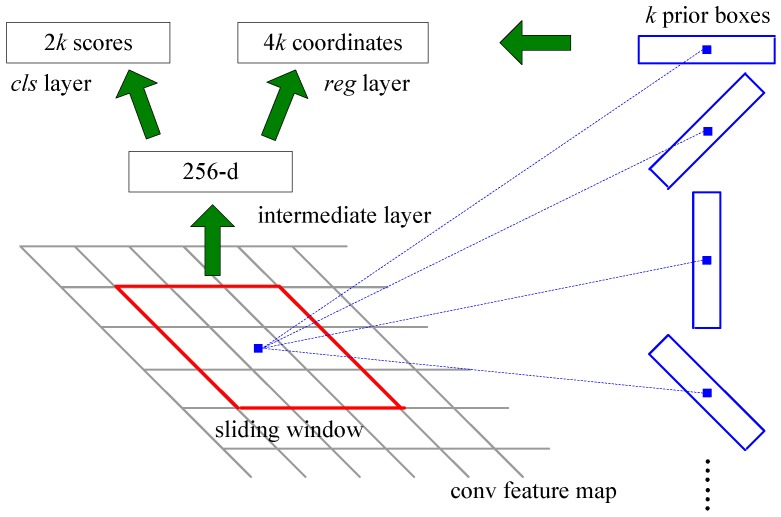
Diagram of target detection part.

The network structure is similar to that of the region proposal network of the Faster R-CNN method [12]. First, we use layers from cov1_1 to conv5_3 of VGG16 [16] to extract the features of the image. VGG16 model is pretrained on the ImageNet dataset [15], which has high performance in object classification. Many methods of target detection are fine-tuned from it, such as Faster R-CNN and SSD. So we choose this model and fine-tune it to get a better performance. Then, based on the feature maps, we design two flow paths with two convolutional layers to simultaneously predict the loss label and regress the location-angle offsets. Finally, we calculate the loss with the loss layer and backpropagation to train the model. The network structure is shown in Figure 3 and the diagram of the detection stage is shown in Figure 4.

### 3.2. Rotated Prior Box

**Figure 5:**
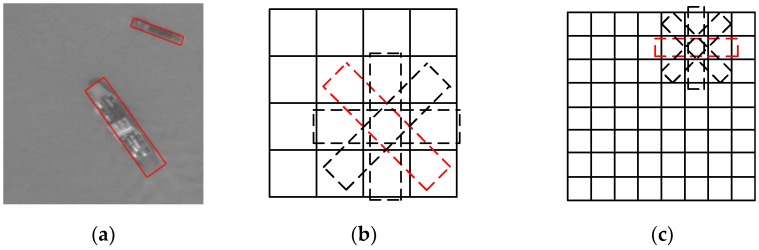
Diagrams of the ground-truth bounding box and the best matching prior box. (**a**) The image and the ground-truth box. (**b**) Prior boxes with dotted line and the best matching prior box with the bigger ship, which is in red. (**c**) Prior boxes with dotted line and the best matching prior box with the smaller ship, which is in red.

After obtaining the convolutional feature maps, we set the rotated prior boxes on each position of the feature maps to locate the ship targets. The rotated prior boxes are shown in Figure 4, which is in blue color. Based on the rotated prior boxes, we can predict the class name and location information of the target in various orientations. When training the network, we first match the prior boxes with the ground-truth bounding boxes to obtain the positive sample information, which will be used to calculate the loss. The diagram of rotated prior boxes are shown in Figure 5, the one matched with the ground-truth bounding box is shown in red.

**Figure 6:**
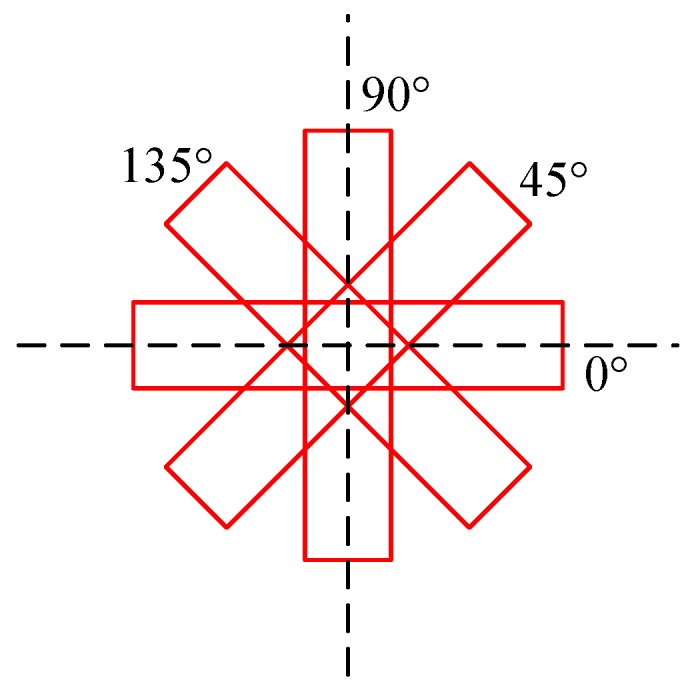
Diagram of the selected angles of the rotated bounding boxes.

After splitting the large-scale image, we feed the network with images of size 600 × 600 pixels. The scale of the prior box is set as {32,64,128,256} and the aspect ratio means the ratio of *h* to *w*, which is set as 5:1, so the long side *h* of the box is {71,142,284,568}; the step size of the box is 16. Using multi-scale prior boxes, we can detect the multi-scale targets better. The rotation angle is set as {0,45,90,135}, which ignores the head or tail direction as it is difficult to identify when the ship scale is small. The rotated prior boxes of one scale are shown in Figure 6. These parameters are chosen based on the characteristics of the ship targets in the dataset and the network structure, which will be explained in details in Section 4.3.

### 3.3. Rotated Box Matching

Rotated box matching plays an important role in target detection, which is used in choosing positive samples in training, nonmaximum suppression in testing, and deciding whether it is the right detection in evaluation. Let us assume two rotated boxes, recorded as $box_{1}$ and $box_{2}$. We use a matrix with all zeros except the ones in the box region to record the box, so we can obtain the area of the box by computing the sum of the matrix elements. The two matrices are recorded as $I_{1}$ and $I_{2}$. The areas of $box_{1}$ and $box_{2}$ are recorded as $Area_{1}$ and $Area_{2}$. To obtain the overlap region, we calculate the product from $I_{1}$ and $I_{2}$. The result is recorded as matrix $I_{3}$, and the overlap area is the sum of the elements, recorded as $Area_{\cap }$. Thus, the matching degree of the two boxes is calculated as

(1) $$matching\;degree=\frac{Area_{\cap }}{Area_{\cup }}=\frac{Area_{\cap }}{Area_{1}+Area_{2}-Area_{\cap }}\;$$

When matching the rotated boxes, the calculation between each prior box will lead to a large amount of calculation. Thus, we should first narrow down the selection scope. We set a selection range $t_{c}$ and calculate the distance between the center points $d_{c}=\left\|\left(x_{1}^{c},y_{1}^{c})-(x_{2}^{c},y_{2}^{c}\right)\right\|$, where $\left(x_{1}^{c},y_{1}^{c}\right)$ and $\left(x_{2}^{c},y_{2}^{c}\right)$ are the center point coordinates of the two boxes. If $d_{c}<t_{c}$, we select a prior box with the nearest angle to the ground-truth box angle to calculate the matching degree. By implementing these strategies the calculation amount is significantly reduced.

### 3.4. Loss Function

There are two flow paths to output the result, one predicts the class information of the target and the other predicts the location and angle information. The two layers are usually called the class label output layer and location angle regression layer. When calculating the loss, we combine the loss of class label output layer and the location angle regression layer to calculate the weighted sum. Using $x_{ij}^{}=\{1,0\}$ to record whether the ith prior box is matched with the jth ground-truth box, 1 means matched and 0 means mismatch. The loss is calculated as

(2) $$L(x,c,l,g)=\frac{1}{N}(L_{cls}(x,c)+\alpha L_{loc}(x,l,g)),\;$$

where x is the matching status, c is class label output, l is the output of location and angle offsets, g is the ground-truth information, and N is the number of prior boxes matched with the ground-truth boxes. If N=0, the loss is 0. $L_{cls}$ is the loss of class label output layer, $L_{loc}$ is the loss of the location and angle offset regression, and α is the weight, which is used to balance the two losses, here we set α to 1 experimentally.

The loss of class label output layer is calculated as

(3) $$L_{cls}(x,c)=-\sum_{i\in Pos}^{N}x_{ij}^{}\log({\hat{c}}_{i}^{1})-\sum_{i\in Neg}\log({\hat{c}}_{i}^{0}),\;$$

(4) $${\hat{c}}_{i}^{1}=\frac{\exp(c_{i}^{1})}{\sum_{p}\exp(c_{i}^{1})},\;$$

where $c_{i}^{}$ is the output of class label layer, ${\hat{c}}_{i}^{}$ is the class score, ${\hat{c}}_{i}^{1}$ is the probability of having a ship target in the box, and ${\hat{c}}_{i}^{0}$ is the probability of being the background. When calculating the weighted sum of the log function, we should see whether it is a positive or negative prior box after matching with the ground-truth boxes.

The loss of regression of location and angle offsets uses the SmoothL1 loss [11] for the calculation:

(5) $$L_{loc}(x,l,g)=\sum_{i\in Pos}^{N}\sum_{m\in \{cx,cy,w,h\}}x_{ij}^{k}{\mathrm{smooth}}_{L_{1}}(l_{i}^{m}-{\hat{g}}_{j}^{m}),\;$$

(6) $${\mathrm{smooth}}_{L_{1}}(x)=\{\begin{array}{cc} 0.5x^{2} & \mathrm{if}\;\left|x\right|<1\\ \left|x\right|-0.5 & \mathrm{otherwise}, \end{array}\;$$

where l is the predicted offsets of the five parameters $\left(x_{c},y_{c},h,w,theta\right)$ and ${\hat{g}}_{j}^{}$ is the offset between the ith prior box $d_{i}^{}$ and the jth ground-truth box $g_{j}$, as shown in Equations (7) and (8):

(7) $${\hat{g}}_{j}^{x_{c}}=(g_{j}^{x_{c}}-d_{i}^{x_{c}})/d_{i}^{w},{\hat{g}}_{j}^{y_{c}}=(g_{j}^{y_{c}}-d_{i}^{y_{c}})/d_{i}^{h}\;$$

(8) $${\hat{g}}_{j}^{w}=\log\left(\frac{g_{j}^{w}}{d_{i}^{w}}\right),{\hat{g}}_{j}^{h}=\log\left(\frac{g_{j}^{h}}{d_{i}^{h}}\right),{\hat{g}}_{j}^{theta}=\tan(g_{j}^{theta}-d_{i}^{theta})\;$$

When calculating the loss, the sum is calculated only if the prior box is matched with a ground-truth box.

### 3.5. Implementation Details

We use Caffe [26] to build the network structure and train our deep neural network. In the training stage, we process one image at one iteration. In the image, we select the prior boxes with a matching degree larger than 0.7 or those having the largest matching degree with a ground-truth box as the positive samples. The positive number $N_{p}$ is not more than 64; the negative samples are randomly selected in the ones with matching degree below 0.3. The negative number $N_{f}$ is $128-N_{p}$, so the total number is 128. We use the stochastic gradient descent method to optimize the training. The initial learning rate is 0.001 and the number of iterations is 80,000. After 60,000 iterations, the learning rate is reduced 10-fold.

In the testing stage, we set the score threshold at 0.25 to decide whether there is a target in the box. Then, we perform the nonmaximum suppression to obtain the final result. We set the nonmaximum suppression threshold at 0.2 to remove the boxes whose matching degree is larger than 0.2 with another box of a larger score.

## 4. Experiments and Analysis

The algorithm was tested on images with ship targets in complex backgrounds to evaluate the detection and localization performance. We tested on a properly selected dataset and compared our algorithm with those using horizontal bounding boxes, such as Faster R-CNN and SSD, to show the advantage of detection using rotated boxes. We also carried out a comparison with existing algorithms using rotated bounding boxes, such as DRBox and OBB Faster R-CNN, to show the robustness of our algorithm.

### 4.1. Dataset

We collected 640 remote sensing images from Google Earth. The long side of the image was shorter than 1000 pixels. There were 4297 ship targets with complex backgrounds in the images. The complex clutter included wave clutter, ships in close proximity, and ships close to the shore.

### 4.2. Performance Evaluation Index

We used the average precision (AP) value to evaluate the performance of the algorithm. AP is the area under the precision-recall curve. Precision is the ratio of correct detection to the total number of targets detected. Recall is the ratio of correct detection to the total number of ground-truth targets. When we change the score threshold, we will get a different precision and recall. The precision–recall curve shows the precision variety following the recall variety, so it can reflect the overall performance of the algorithm. When considering whether it is a correct detection, we calculated the matching degree between the detected box and the ground-truth box, as in Equation (1). If the matching degree is higher than 0.5, we consider it as a correct detection. When a ground-truth bounding box has been matched with a detected box, then other detected boxes matched with it are considered as false alarms.

### 4.3. Performance of the Algorithm and Parameter Analysis

As the ships vary in scale and appearance, the algorithm should select suitable multiscale parameters and augment the data to learn more about the variety.

#### 4.3.1. Dataset Characteristics Analysis and Model Parameter Selection

First, we analyzed the characteristics of the ship targets in the dataset to help select the model parameter. We recorded the long side of the rotated box as h, the short side as w, the aspect ratio as h/w, and the angle as theta.

**Figure 7:**
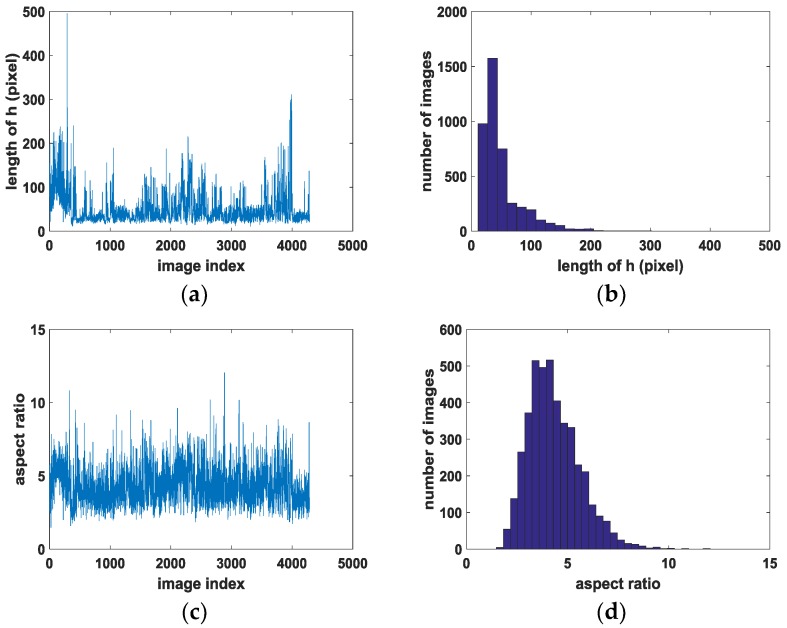

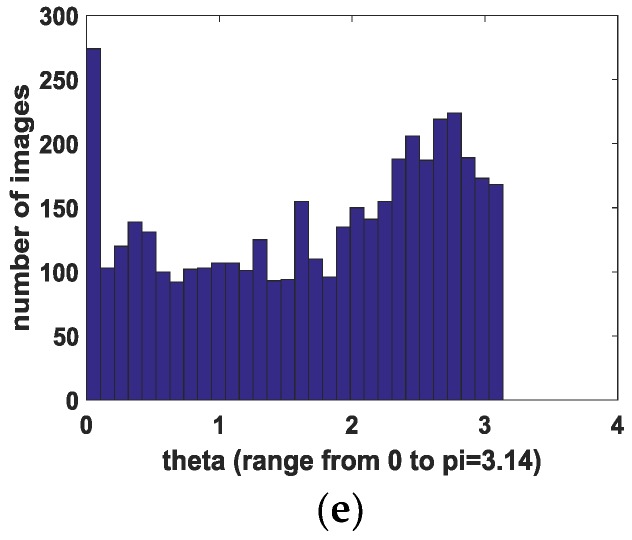
Main characteristics distribution of the targets in the dataset. (**a**) The long side h of all 4279 targets, (**b**) distribution of long side h, (**c**) aspect ratio h/w of all 4279 targets, (**d**) distribution of aspect ratio h/w, and (**e**) distribution of angle theata.

Figure 7 shows the main characteristics distribution of the targets in the dataset. The long side h is distributed mainly from 20 to 150 pixels. The longest is about 500 pixels, while others are distributed between 200 and 300. The mean length is 50.6, and the aspect ratio h/w is distributed mostly between 3 and 7. The largest value is 12, while others are distributed between 8 and 10. The mean value is 4.3. The angle of the ships is mainly distributed around 0° and uniformly distributed at other angles.

After analyzing the characteristics of the targets in the dataset, we can use them to help select the parameters. We set the aspect ratio to 5 so that the rotated prior boxes can match the ground-truth boxes well. When choosing the scales of the prior box, we consider the long side h to be mainly distributed between 20 and 150 and the larger ones to be mainly between 200 and 300. Therefore, we select the scales at {32,64,128,256} to better match the characteristics of the real targets when the shorter side of the input image is bigger than 600 pixels and the longer side is smaller than 1000 pixels and is not preprocessed. When performing data augmentation, we rotate the images and label information to increase the variability of the targets. Since the size of the input image to the deep network is limited to 600–1000 pixels, the images with a larger or smaller scale will be preprocessed and the target scale is changed. Therefore, we should add zeros to the small images to expand them and split them with a large scale to retain the original size of the target.

#### 4.3.2. Model Parameter Analysis

We trained the deep network with different parameters and compared the performances to analyze the influence of the parameters.

We trained on a dataset of 148 images with 80,000 iterations and an aspect ratio of 3. The performance AP was 0.091 when testing on all 640 images. When changing the aspect ratio to 5 and retaining all other parameters, the performance AP remained at 0.091. When the training dataset contained 200 images and the aspect ratio was 5, the performance AP increased to 0.212. These results show that training with less data will not help learn the ship’s feature well, resulting in poor performance. When training with 200 images, the algorithm is more robust because a closer distribution of the training and testing datasets.

Then, we considered the influence of input image scale and preprocessed the images to keep the target within the original scale. After the preprocessing, we trained with a training dataset of 400 images with 80,000 iterations and an aspect ratio of 5. This caused the performance AP to increase to 0.362. The result shows that having more training samples and maintaining the original target scale will help the algorithm to learn more robustly and obtain better performance. Performances with different parameters are shown in Table 1.

### 4.4. Performance Comparison with Algorithms Using Horizontal Bounding Boxes

The commonly applied methods in the target detection area use the horizontal bounding boxes to search and detect targets, such as SSD and Faster R-CNN. Here, we compare the performances of the two algorithms with our method to show the advantage of using rotated bounding boxes.

**Figure 8:**
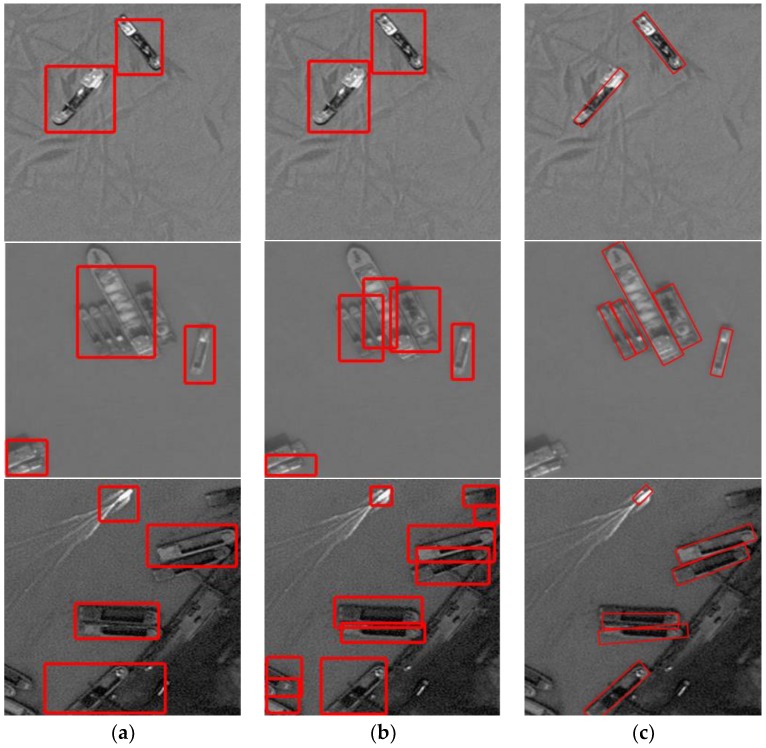
Comparison with the algorithm using horizontal bounding boxes. (**a**) Detection results of SSD, (**b**) detection results of Faster R-CNN, and (**c**) detection results of our method.

We trained on a dataset with 400 images using an SSD algorithm and tested all 640 images. In this case, the performance AP was 0.206. While using the Faster R-CNN algorithm with the same parameters, the performance AP is 0.293. In our algorithm, the performance AP was 0.362. These results show that the method using rotated bounding boxes can learn the target characteristics better and detect the rotated ships in close proximity better. Figure 8 presents a comparison of the detection results between algorithms using horizontal bounding boxes and our method. These results show that both the method using horizontal boxes and that using rotated boxes can detect the ships off-shore well. When the ships are in close proximity or close to the shore, our method using rotated bounding boxes can detect the target better and locate it more precisely. A comparison of the performance AP values is shown in Table 2.

### 4.5. Performance Comparison with Other Algorithms Using Rotated Bounding Boxes

We compared the performance of our algorithm with that of algorithms using rotated bounding boxes to show the advantage of our method. One of the methods is DRBox, which is based on SSD and combined with rotated bounding boxes to detect the oriented targets. We used the trained model provided by the author to test on all 640 images and found the performance AP to be 0.241. The detection results indicated that the model detects large-scale ships better while many small ships were missed. Hence, we added a larger resolution scale to detect the smaller ships and the performance AP was found to increase to 0.305. We continued by adding another larger resolution scale, and the performance AP dropped to 0.277. These results indicate that both the correct detection number and false alarm number are increasing. Overall, the performance of DRBox algorithm is lower than that of our method since the accuracy of detecting small targets is low and more false alarms are detected. Another method is the oriented bounding box (OBB) Faster R-CNN algorithm, which uses a rotated bounding box with eight parameters to detect the oriented targets. We tested the provided trained model on all 640 images and found the performance AP to be 0.278, which is also lower than that of our method. The main reason leading to the performance drop is the missing of several small ships.

**Figure 9:**
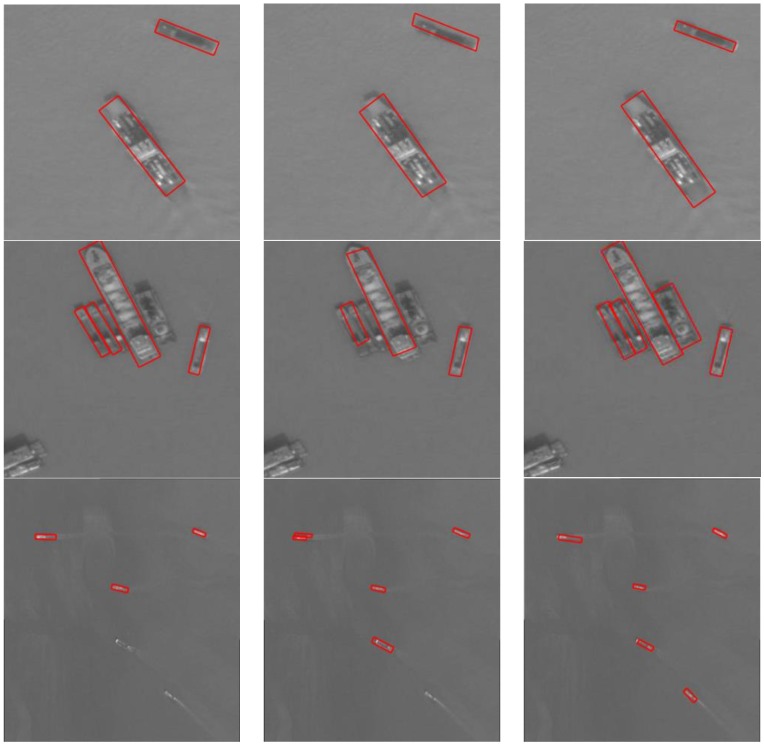

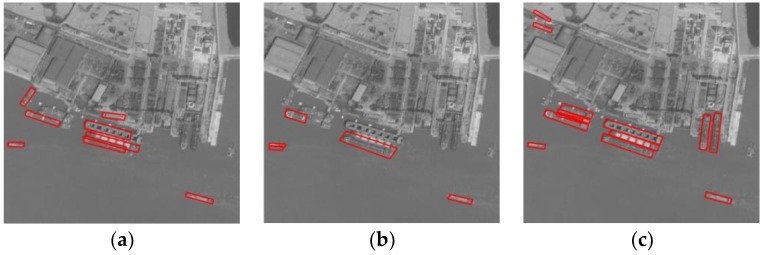
Comparison with other algorithms using rotated bounding boxes. Detection results of (**a**) DRBox, (**b**) OBB Faster R-CNN, and (**c**) our method.

Figure 9 shows that when detecting ships with a background of water, all three algorithms can detect well and locate the ships precisely; when the ships are in close proximity or close to the shore, our algorithm detects better, while other methods miss some ships; when detecting small ships, our algorithm performs better, while other methods miss some ships. The last column shows some false alarms on the land background in both DRBox method and our method, while the OBB Faster R-CNN method misses some ships. The performance AP values of the different methods mentioned above are shown in Table 2.

### 4.6. Discussion

The above experiments and analysis show that our method using multiscale rotated bounding boxes can detect the ships and locate precisely when the ships are in complex backgrounds. Compared with the method using horizontal bounding boxes, our method detects better when ships are in close proximity or close to the shore; compared to other methods using rotated bounding boxes, our method detects ships with higher accuracy and lower missing detection rate, and especially detects small ships better. However, there are still some problems, such as false alarms; as a result, the performance AP still has room for improvement.

## 5. Conclusions

We proposed a multiscale rotated bounding box-based deep learning method to detect oriented ships in complex backgrounds in remote sensing images. The algorithm used five parameters to determine a rotated bounding box, which limits the box to a rectangular shape. The algorithm predicted different positions of the feature maps extracted by a pretrained deep network to output the class label and location-angle offset regression based on the multiscale rotated prior boxes. In the training stage, we searched the best-matched prior boxes with the ground-truth boxes to obtain the positive sample information, which was used to learn the ship features. When matching the rotated boxes, we first narrowed down the search scope to reduce the amount of calculation. In the testing stage, we input the testing image to the network and perform forward calculation to obtain the class labels and the location-angle offsets of each position. Then, we set a threshold of the class score to determine whether it is a ship and perform post-processing, such as nonmaximum suppression, to obtain the final detection result. The experimental result showed that our method can detect ships robustly under complex conditions, such as ships in wave clutter, in close proximity, close to the shore, and with different scales, and obtain an accurate location of the ships. Our method performs better than the other methods. However, the performance AP still has room for improvement, so we will study the target missing and false alarm problems in the future to improve the algorithm.

## Figures and Tables

**Table 1:** Performance comparison of our method with different parameters.

Number of Training Samples	Aspect Ratio	Keep Original Scale	AP
148	3:1	no	0.091
148	5:1	no	0.091
200	5:1	no	0.212
400	5:1	yes	0.362

**Table 2:** Performance comparison of different algorithms.

Method	AP
SSD	0.206
Faster R-CNN	0.293
DRBox	0.305
Faster R-CNN (OBB)	0.278
Our method	0.362
